# Ultrasound-Stimulated PVA Microbubbles for Adhesive Removal from Cellulose-Based Materials: A Groundbreaking Low-Impact Methodology

**DOI:** 10.1021/acsami.1c01892

**Published:** 2021-05-14

**Authors:** Alessia D’Andrea, Leonardo Severini, Fabio Domenici, Sultan Dabagov, Valeria Guglielmotti, Dariush Hampai, Laura Micheli, Ernesto Placidi, Mattia Titubante, Claudia Mazzuca, Gaio Paradossi, Antonio Palleschi

**Affiliations:** † Department of Chemical Science and Technologies, University of Rome “Tor Vergata”, Via della Ricerca Scientifica 1, 00133 Rome, Italy; ‡ INFN-LNF, XLab Frascati Via Enrico Fermi 54, 00044 Frascati (RM), Italy; § University Guglielmo Marconi, Via Plinio 44, 00193 Rome, Italy; ∥ RAS P.N. Lebedev Physical Institute, Leninsky pr 53, 119991 Moscow, Russia; ⊥ National Research Nuclear University MEPhI, Kashirskoe Sh. 31, 115409 Moscow, Russia; # Department of Physics, Sapienza University of Rome, P.le Aldo Moro 2, 00185 Rome, Italy

**Keywords:** polymers, ultrasound, fluorescence, gels, microbubbles, poly(vinyl alcohol), cultural heritage

## Abstract

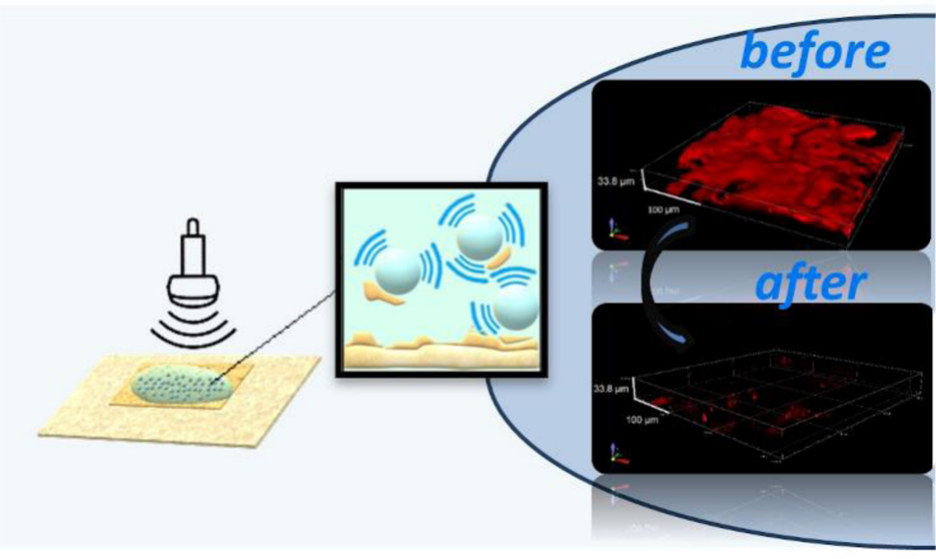

In this work, we shed new light on ultrasound contrast agents applied to the field of cultural heritage as an invaluable fine-tune cleaning tool for paper artworks. In this context, one of the primary and challenging issues is the removal of modern adhesives from paper artifacts. Modern adhesives are synthetic polymers whose presence enhances paper degradation and worsens its optical features. A thorough analytical and high-spatial-resolution combined study was successfully performed to test the capability of poly(vinyl alcohol)-based microbubbles stimulated by a proper noninvasive 1 MHz ultrasound field exposure in removing these adhesives from paper surfaces, in the absence of volatile invasive and toxic chemicals and without damaging paper and/or leaving residues. We demonstrate that poly(vinyl alcohol)-shelled microbubbles are suitable for interacting with paper surfaces, targeting and boosting in a few minutes the nondamaging removal of adhesive particles from paper samples thanks to their peculiar shell composition together with their ultrasound dynamics.

## Introduction

Nowadays, microbubbles find a well-established place in routine medical diagnostics.^1−6^

In this context, we address the multifunctional use of microbubbles by coupling diagnostics with therapeutic treatment to accomplish an efficient theragnostic device. At present, there is still enough room to choose different materials for the core and shell of microbubbles to adjust their features, while preserving the high acoustic impedance that characterizes any type of microbubbles. In this respect, two main classes of microbubbles, having either lipid or polymer shells, are currently being investigated as ultrasound contrast agents (UCAs). Such diverse materials exhibit differences in the resonance frequency and in the stability of the microbubbles. Polymer-shelled microbubbles last longer once injected in the bloodstream than lipid ones, but they usually resonate at higher frequencies exhibiting worse or comparable echogenicity to that of the lipid-shelled ones.^3^ Despite their great potentiality, the use of microbubbles in the field of cultural heritage for the conservation and restoration of artworks based on biopolymers such as paper or other degradable materials is, to date, almost unexplored.

Paper is the main writing support on which historical events and fundamental conquests of humankind have been reported in the past and where they are still registered in the present day. Books and documents stored in libraries, museums, or archives are at risk of loss due to their inherent fragility. The main component of paper, cellulose, is subject to irreversible and spontaneous degradation processes, which are accelerated by environmental factors like humidity, temperature, pollution, and radiation. Degradation is mainly due to acidic hydrolysis and oxidation, two strictly related processes (oxidation leads to acid byproducts, which, in turn, trigger hydrolysis) that cause mechanical fragility and decline of the optical quality of paper samples.^7,8^ In addition, the presence of other materials such as adhesives or clips further enhances paper degradation, leading to yellowing, foxing, and acidity increase, thus shortening the document’s lifetime.^9^ Normally, the preservation of paper artworks involves the wet removal of degradation byproducts and pollution with the aim of slowing down aging processes. The common procedure includes immersion in water, which can cause irreversible damages to paper substrates and inks, colorants, or pigments. Several strategies have been developed to avoid these consequences, such as the use of hydrogels from natural or synthetic sources.^10−13^ Hydrogels offer several advantages over water baths: thanks to their water retentive properties, they are able to release water and absorb byproducts and other contaminating compounds by capillarity. Furthermore, gels can remove dust and solid contaminants via interfacial adhesion.^14,15^ Their use has allowed the restoration of very fragile paper samples and the removal of specific materials like natural adhesives or greasy compounds.^11,16−19^ Despite the great development of cleaning hydrogels, there are still several drawbacks. The first is related to their rigidity, making it difficult or even impossible to conform to rough, fibrous surfaces to penetrate paper pores and interact with the interior fibers of paper: their action is limited to water release and uptake processes. Second, their action is relatively time-consuming (about 1 h).^10,16^ Despite the great attention addressed to the cleaning of ancient paper artworks, very little has been done on this issue for modern paper crafts, even if they are more fragile and tend to degrade faster, as they are composed of mechanically and/or chemically treated wood pulp instead of rags.^20,21^ In this context, the characterization of modern paper, as well as the setting up of suitable procedures for their wet restoration, is almost unexplored.^10,11,20,22^ Recent articles outline the complexity and reduced efficacy of the cleaning procedure on modern paper with respect to ancient paper.^12,23^ Furthermore, the removal of foxing due to modern adhesives to the paper surface is an almost unexplored field.^9,24,25^ Tapes were extensively used since their discovery to repair broken parts of paper, but, with time, paper in contact with them tends to become brittle and brown/yellow independent of the adhesive composition. The very high degradability to which they are subject makes their removal mandatory, after some time, which could be very difficult to obtain through traditional restoration and conservation procedures. One of the crucial points in this field is indeed the removal of residues of aged synthetic adhesives (pressure-sensitive adhesive tapes, PSATs) from paper sheets. In the literature, to the best of our knowledge, very few articles regarding the characterization and removal of modern aged adhesives are present, and unfortunately, they all involve organic solvents, harmful for both restorers and artworks due to their chemical aspecificity.^9,25^

The use of multicomponent biocompatible surfactants revealed to be an efficient alternative cleaning strategy, but it may present critical steps in the preparation and treatment (e.g., complex preparation, organic solvents in the form of nanostructured fluids can break during treatment).^9,24,26,27^

To complicate the scenario, one of the more stringent requests for an innovative restoration procedure is the absence of residues released by the cleaning materials on the artwork. This is because residues can modify the overall features of the artwork from historical and chemical points of view and can induce long-term damages. Therefore, assessing the removal of cleaning materials after treatment is of paramount importance, as well as establishing that the proposed strategy does not damage, even slightly, the paper sample.

In this article, we propose, for the first time, the combined use of microbubbles and ultrasounds (US) for the selective removal of a coating from a delicate substrate.

In particular, we present a new and relatively easy-to-use method to clean modern paper from adhesives in a rapid and noninvasive manner. The idea comes from the finding that successful cleaning needs an intimate contact of the material employed with both the substrate and compounds to be removed, as observed by comparing the times needed for a complete cleaning process with gellan hydrogel (1 h) or with the corresponding gellan microgels (few minutes).^10,14,15,23^ It has been demonstrated that poly(vinyl alcohol) (PVA)-based hydrogels are good materials for cleaning both ancient and modern paper samples.^12,13^ In addition, PVA-shelled microbubbles are very stable and acoustically active, thus representing a versatile tool to enhance the cleaning action with US.^28,29^ On this basis, we defined a novel strategy for the removal of adhesives from modern paper that combines short-term treatment based on PVA-shelled microbubbles and US treatment, followed by a final cleaning step with hydrogels made up of PVA and telechelic PVA (tel*-*PVA; see Scheme S1).^12,13^ This is an innovative approach, which makes use of microbubbles coupled with ultrasounds for the selective cleaning of coating from paper. It should also be pointed out that the deployment of US in the absence of other supporting devices such as microbubbles, for diagnostic or cleaning purposes on cultural heritage, has been reported only in a few cases^30−32^ involving artworks, and no results have been published concerning paper artworks. To assess our idea, we characterized the interaction of PVA microbubbles (herein PVAMBs) with paper and their cleaning efficacy using several experimental techniques, such as confocal fluorescence microscopy, attenuated total reflectance–Fourier transform infrared (ATR-FTIR) spectroscopy, X-ray diffraction (XRD), visible reflectance spectroscopy, and tensile tests. Preliminary investigations concerning a suitable cleaning protocol are also reported.

## Experimental Methods

### Materials

PVA (fully hydrolyzed), NaIO_4_, HCl, fluorescein isothiocyanate (FITC), methanol, and dimethyl sulfoxide (DMSO) are from Merck (Merck KGaA, Darmstadt, Germany). All reagents were of analytical grade and used without further purification. Double-distilled water (Millipore, Billerica, MA) was used for the preparation of all solutions. Modern paper samples were from a 1994’s commercial, nonsatinated notebook paper (grammage: 69 g/m^2^; Blasetti, mod. Clarissa Maxi, Blasetti, Pomezia, Italy) from a private collection source. PSAT pieces were from the same notebook, where they were used to repair paper tears and goats.

### PVAMBs Synthesis and Characterization

The PVAMBs were prepared according to a previously reported protocol.^29,33^ In detail, 4 g of PVA was dissolved in 200 mL of Milli-Q water. The solution was then stirred on a heating plate at 80 °C until PVA solubilization occurred. Then, 0.4 g of sodium metaperiodate was added. After 1 h, the solution was cooled to room temperature and then stirred for 2 h at 8000 rpm (UltraTurrax, IKA, Germany) to form PVAMBs from the embedded air through agitation. The so formed PVAMBs were then washed extensively for several days through a separatory funnel against double-distilled water to remove unreacted reagents. The suspension was then centrifuged at 1000 rpm for 10 min, many times until the PVAMBs dispersion does not present impurities or PVA residues, to isolate the supernatant containing the PVAMBs (generally 5–10 centrifugations are necessary). A small amount of starting solution was diluted 1:2. About 10 μL was were put on a Neubauer counting chamber, and the microbubbles were counted under a microscope with a 40× objective using ImageJ freeware for the analysis. The final concentration was estimated to be about 2 × 10^8^ PVAMBs/mL. After each preparation of PVAMBs, the acoustic characterization was also performed as measured by attenuation spectroscopy (not shown) confirming that the acoustic resonance, viscoelastic, and thickness features of the PVAMBs shell are well reproducible compared to the values in the literature.^28^

To label PVAMBs with a fluorescent probe, 50 μL of FITC (5 mg/mL in DMSO) was added to 5 mL of starting solution. After 1 h of stirring in the dark, the excess fluorophore was removed by centrifugation. The mean diameter of the PVAMBs and their distribution was assessed by confocal microscope (Nikon, Florence, Italy) and dynamic light scattering (DLS) measurements (Brookhaven, New York). This instrument is equipped with a BI-200SM goniometer and a solid-state laser, which emits radiation at 532 nm. The experiments were carried out at room temperature. The correlation function of the scattered intensity was analyzed with the algorithm CONTIN included in the software of the instrument.

### PVA Hydrogel Synthesis

PVA hydrogels were prepared as previously reported.^34^ The hydrogel contains 5% of PVA and 10% of tel-PVA. In detail, tel-PVA was prepared by adding 2% (mol/mol of PVA repeating units) of solid NaIO_4_ to a PVA solution, to allow the complete oxidation of the head-to-head PVA sequences at 60 °C. After 20 min, the solution was cooled to room temperature. An aqueous PVA solution at about 80 °C was then added to the tel-PVA solution, and the system was acidified at pH = 2.0 with HCl. The mixture was left for 24 h in the reaction vessel to complete the cross-linking of the polymer chains and gel formation. The gels were then exhaustively washed with double-distilled water for several days until the conductivity of water was about 1 μS, and no PVA traces were detected, by means of ATR-FTIR analysis on dried residues of the water used for the washings.^13^

A picture illustrating the difference in the PVAMBs and PVA hydrogels is reported in Scheme S1.

### Paper Sample Characterization

#### Paper Composition

Paper fiber composition was estimated by exposing them to Graff “C” stain.^35^ Graff “C” solution was prepared by mixing in 52 mL of ZnCl_2_ saturated solution, 0.06 mol of AlCl_3_, 0.06 mol of CaCl_2_, 0.64 mmol of I_2_, and 1.4 mmol of KI. A drop of stain was applied to a very small portion of each sample, previously chopped with the help of a droplet of water. The sample was then placed on a microscope slide and observed with a Nikon Eclipse Ti-E microscope with a 20× objective.

#### Adhesives Removal

After manual removal, with the help of tweezers, of the adhesive backside, the area to be treated was fully covered with a PVAMBs dispersion (*V* = 400 μL; concentration = 2 × 10^8^ PVAMBs/mL on a sample area of about 2 × 2 cm^2^). A US pulse was then applied on the area of interest for 2 min (Sonidel SP100 Sonoporator; Sonidel Limited). A duty cycle of 100% and intensity of 5 W/cm^2^ and 1 MHz of frequency were set. In detail, the cleaning process was carried out manually, rotating the tip of the probe on the PVAPMBs dispersion on the adhesive/paper to be cleaned. Temperature before and after the treatment was monitored using a tip thermometer HI151 Checktemp 4, 0.1 °C accuracy (Hanna Instruments, Italy). The treatment was performed at room temperature (20.0 °C); the final temperature did not exceed 35.0 °C and dropped after the US was switched off. To ensure the removal of PVAMBs from the paper surface, a piece of PVA gel was used as a soft sponge, dabbing for a few seconds, paper sample with it. The dabbing was repeated at least five times for each sample.

PVA hydrogel alone has been applied according to the procedure reported earlier.^13^

#### Fluorescence Confocal Microscopy

Paper samples were analyzed before and after the treatment. Small samples (about 2.0 cm^2^) were cut from the sheets of paper and put on a microscope slide; then, some drops of water were added to wet the paper, to better focus the sample. A coverslip was then added and fixed over the sample, and the specimen was analyzed with a Nikon C1 microscope equipped with two lasers (the first one with argon ions and λ_excitation_ = 488 nm, the second one with helium–neon and λ_excitation_ = 543 nm). Fluorescence was observed in both the green and red channels, and it was attributed to the residual adhesive. Three-dimensional (3D) pictures were acquired with the same instrument.

3D images were composed of 225 consecutive *z*-series of two-dimensional (2D) images collected using a 40× objective and a *z*-axis step of 0.15 μm. The 3D image was generated with the *xyz* dimensions of 318 × 318 × 33.8 μm^3^. Images were acquired using both excitation lasers.

#### Atomic Force Microscopy (AFM)

The morphology of samples was studied by a Veeco Multiprobe AFM (Nanoscope IIIa); 20 × 20 μm^2^ images were acquired in contact mode by means of the same V-shaped Si_3_N_4_ tip with a stiffness of 0.32 N/m. The images were analyzed by Gwyddion software.^36^

#### X-ray Diffraction (XRD) Analysis

The structure and crystal orientation of the samples were studied by X-ray diffraction (XRD) measurements performed by an XRD 3003 Seifert θ/2θ diffractometer. This instrument is a 2200 W power system with a Cu Kα anode target, 1 × 12 mm^2^ beam dimension, and 0.001° angular resolution. The XRD pattern was obtained using a tension of 40 kV, a current of 30 mA, an acquisition time of 1 s/step, and an angular scan of 0.02°/step.

#### ATR-FTIR Experiments

FTIR spectra were recorded with an is50 instrument (Thermo Scientific, Inc., Madison WI), equipped with a single-reflection ATR diamond cell. Measurements were performed in the 4000–525 cm^–1^ region, at a resolution of 4 cm^–1^. A total of 32 scans were collected for each measurement. Spectra were collected by placing the samples directly on the ATR cell.

#### High-Performance Liquid Chromatography (HPLC) Analysis and pH Measurements

HPLC analysis was performed with a THERMOQUEST instrument (Shimadzu, Kyoto, Japan), equipped with two pumps and an ultraviolet/visible (UV/vis) detector LCGA SPD-10A (Shimadzu, Kyoto, Japan). A chromatographic column HPLC Pinnacle II C18, 5 μm, 250 and 4.6 mm (RESTEK) was used. The chromatographic analysis was performed on extracts obtained by soaking 1 cm^2^ of every sample with 1.5 mL of methanol, stirring on the rotating wheel (Dynal AS, Sweden) overnight at room temperature. Further experiments were performed extracting the adhesive residues from the paper samples in 1.5 mL of water (stirring overnight at room temperature). This method of extraction was chosen to facilitate the extraction of the organic acids arising from the process of paper degradation. Analyses of the samples extracted in water were carried out under isocratic conditions using 25 mM phosphate buffer at pH 2.4 and 10% (v/v) methanol as a mobile phase, while those extracted in methanol were performed using a 40:60 (v/v) water/methanol mixture with 0.05% of 99% formic acid, at pH = 3.0. In the last case, an internal standard (retention time: 3 min) was used. The chromatographic conditions were chosen to provide evidence of the compounds characterized by low-molecular-weight and hydrophilic behavior (e.g., cellulose degradation byproducts and PVAMBs). The flow rate was 0.8 mL/min, with a loop of 200 μL and detection wavelength λ = 230 nm. The analyses were performed before and after the application of PVAMBs.^10,23^

Measurements of pH were carried out on the paper surface using an Amel pH-meter 334-B pH-meter with a combined glass microelectrode Ag/AgCl and a porous PTFE diaphragm (Crison Instruments, Spain). At least three measurements were performed for each sample.^37^

#### Chromatic Variation Measurements

Measurements concerning the optical quality of paper were performed using a Konica Minolta CR-200. Coordinates in the CIELAB color space (L*, a*, b*) were obtained using a D65 illuminant and a 10° observer. Chromatic variation tests before and after cleaning were reported in terms of Δ*E*, which is the distance between two points in the CIELAB space. Results were obtained from three measurements on the same spot.^13^

#### Tensile Tests

Tensile tests were performed on paper specimens with dimensions 200 × 15 mm^2^, using the universal testing machine (Lloyd LRX) equipped with a load cell of 50 N. A gauge length of 80 mm, a crosshead speed of 5 mm/min, and a preload of 0.2 N were set following the UNI EN ISO 1924-2:2009 standard.

## Results and Discussion

The investigation of PVA-shelled microbubbles brought up several unique features of this US active system. Different from the commercially available lipid-shelled MBs, PVA-based MBs are very stable and can be reconstituted in distilled water from the freeze-dried sample.^29^ This represents a great advantage, allowing us to simplify field work through a US portable generator, thus enabling in situ cleaning processes. PVAMBs herein employed have a resonance frequency around 10 MHz as detected by attenuation spectroscopy. The size distribution of these microbubbles is centered at 4.4 ± 0.4 μm, as determined by confocal microscopy and dynamic light scattering (Figure S1). According to Domenici et al.,^38^ the elastomeric PVA shell confers to the PVAMBs a stiffness comparable to that of lipid-shelled UCAs (i.e., gaseous sulfur hexafluoride core with saturated diacyl phospholipids monolayer shell) and, unlike the latter, they maintain long-term stability in the structural and echogenic properties also under repeated US stimuli. More importantly, cross-linked PVA materials have been recently proven to be optimum candidates for paper cleaning applications.^12,13^ Paper stripes from a modern notebook showing yellow and brittle adhesive residues, dated back to 1994, have been selected for use in a case study, with the purpose of demonstrating the capability of the proposed system to work on an almost unexplored type of paper. The present study started from the characterization of the paper support and its conservation state. In this case, paper has been obtained by bleaching chemically treated wood pulp (through the so-called Kraft process) and thus contains depolymerized cellulose fibers and a very small amount of lignin. This paper-making procedure leads to a fragile, degradable, and easily oxidizable paper.^20,21^ Confirmation of wood pulp processing of the paper fibers has been obtained by analysis with Graff “C” stain (Figure S2): after staining, fibers appeared light bluish-gray or gray, thus implying a low level of lignin in samples, very common for office modern paper samples.^16,39^ Based on the ATR-FTIR spectrum (Figure S3), it was deduced that the adhesive is an aged synthetic rubber (styrene and isoprene copolymer) on a polypropylene or poly(vinylchloride) backing.^40^ With time, this compound is subject to strong oxidation able to modify its chemical composition and to worsen its macroscopic features. Studies on accelerated aging of polystyrene and copolymers containing polystyrene^41,42^ indicate that the oxidation of PS leads to the formation of products (such as α,β-unsaturated aldehydes, saturated ketones, and saturated aldehydes). These groups contribute to the broadening and increasing of the intensity of some diagnostic FTIR bands (like those ascribed to C=O stretching of aldehydes, carbonyl, and carboxyl groups at about 1730 cm^–1^), i and bands assigned to stretching C–O in ether groups, in the 1200–1000 cm^–1^ region).

### Identification of the Most Efficient Cleaning Protocol

The most suitable cleaning procedure has been performed by comparing ATR-FTIR spectra and the chromatic variation before and after every chosen treatment (Table 1). To monitor the cleaning efficacy by comparing the FTIR spectra analyzing the adhesive and paper spectra (Figure S3), we took into account the absorbance ratio (called “adhesive ratio”, AR) between two specific peaks centered at 1024 cm^–1^ and at about 1730 cm^–1^. The 1024 cm^–1^ peak is mainly related to the CO and CC stretching and CCH and OCH bending modes of cellulosic units,^13,43^ while the 1730 cm^–1^ peak is assigned to the stretching mode of the carbonyl moiety of the adhesive.^40,41^ It should be noted that this ratio underestimates the effective removal of the adhesive since both the adhesive and the paper contribute to the absorption band at 1024 cm^–1^; nonetheless, the lower the ratio, the more adhesive is present on paper. Therefore, an increase in this ratio after cleaning indicates that adhesive has been removed.

**Table 1:** Spectroscopic Parameters to Evaluate the Efficacy of the Cleaning Procedures under Investigation^a^

treatment	AR	ΔL*	Δa*	Δb*	ΔE*
untreated	1.8 ± 0.2				
water and US (2 min)	2.8 ± 0.3	0.6 ± 0.2	–0.05 ± 0.02	–1.2 ± 0.2	1.3 ± 0.2
PVA gel alone (60 min)	3.3 ± 0.1	3.6 ± 0.7	1.5 ± 0.1	13.8 ± 0.2	14.3 ± 0.3
PVA gel alone and US (2 min)	3.6 ± 0.2	1.0 ± 0.8	–0.12 ± 0.01	–8.9 ± 0.1	9.0 ± 0.4
immersion of PVA gel on PVAMBs, application on paper, US (2 min), and dabbing with PVA gel	3.9 ± 0.1	1.5 ± 0.9	–0.10 ± 0.02	–9.6 ± 0.1	9.7 ± 0.3
PVAMBs solution directly on paper, US (2 min), and dabbing with PVA gel	9.1 ± 0.3	8.0 ± 0.8	–0.17 ± 0.01	–15.0 ± 0.2	17.0 ± 0.3
without adhesive		9.3 ± 0.8	–0.15 ± 0.01	–0.15 ± 0.01	19.4 ± 0.4

a Adhesive ratio (AR) and position variation of samples in the CIELAB space (on the L*, a*, and b* axes and as a total chromatic variation, i.e., ΔE*) after cleaning (untreated paper has been used as a reference).

As reported in Table 1 and shown in Figure S4, this ratio increases from AR = 1.8 in the uncleaned sample to AR = 3.3 in samples cleaned by applying the PVA hydrogel, for 1 h as reported in the literature.^12,13^

**Figure 1:**
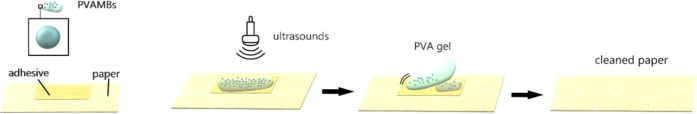
Illustration of the more efficient adhesive removal.

A further increase up to AR = 3.9 has been obtained applying US for 2 min (duty cycle, 100%; intensity, 5 W/cm^2^; frequency, 1 MHz) on PVA gel with PVAMBs. Similar results have been obtained also applying US for 2 min on PVA gel without PVAMBs (data not shown). A much larger increase (5 times higher) of AR ratio (AR = 9.1) has been reached adding PVAMBs directly on the paper sample. US were applied for 2 min, and PVAMBs were then removed by gently dabbing with PVA gel patches (Figure 1). These results demonstrate that the latter method is the most efficient in removing the adhesive. The application of US for 2 min on only water added on paper does not lead to a significant increase in the AR ratio and in the optical quality of paper, thus demonstrating the need of PVAMBs to achieve effective paper cleaning.

**Figure 2:**
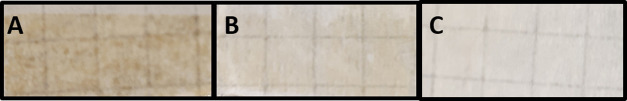
Images (obtained under visible light) of the paper sample before (A) and after treatment (B); paper sample without adhesive (C).

Measurements of the chromatic variation shown in Table 1 corroborate the results obtained by FTIR spectroscopy. An improvement of the optical quality of samples was in fact obtained in all cases, according to the change of position in the CIELAB space after cleaning.^12,13^ More in detail, an increase in brightness (positive L* variation), a decrease in the red tones (negative changes of a* values; except for the treatment with gel alone), and a strong decrease in the yellow component (large shifts on the b* axis) were observed. The largest change in color (ΔE* = 17.0) was obtained by cleaning the paper sample with PVAMB solution applied directly on it, treated with US for 2 min, and then dabbed with PVA gel patches to remove PVAMB residues (Figure 1). The images in Figure 2 clearly highlight the effective removal of adhesives under these conditions; after treatment, the adhesive-coated part of the paper sample was in fact almost indistinguishable from the uncoated part. It should be noted, from Figure 2 (recorded under visible light), that the notebook lines were not removed or faded out by the cleaning procedure.

This has been assessed by ATR-FTIR and chromatic variation data reported in Table 1. In fact, the application of the PVA gel for 1 h on the sample containing yellow adhesive is less effective than the application of PVAMBs for 2 min. The use of US with PVA gel alone for 2 min is a cleaning method as effective as that involving the application of the gel alone for 1 h. Curiously, the use of PVAMBs on PVA gel does not improve the cleaning ability of the system. PVAMBs are not effective if the gel mediates their action, probably because in the PVAMBs adhered onto the PVA gel surface, the gel prevents PVAMBs from entering intimately in contact with paper. This highlights the importance of using PVAMBs in water dispersion to enhance the interfacial interactions of the PVAMBs shell and the adhesive. Recently reported researches, indeed, agree with the idea that cleaning procedures, to be effective (also in terms of time and costs), should involve a close contact between the species used for the removal and the molecules to be removed.^15,23,44^

Moreover, besides achieving such intimate contact, due to the micrometric dimension (Figure S1), compatible with paper roughness (RMS roughness values of paper without or with adhesive are ∼630 and ∼460 nm, respectively; see Figure S5), water, which is a nonsolvent of the pollutant, is used. It allows the transmission of US and conveys the transport of the PVA shell microbubbles as well as the removal of the adhesive given by the PVAMBs dynamic obtained through US application.

It is also worth stressing here that solvent-based paper cleaning strategy undoubtedly allows nanosized agents (e.g., surfactants and nanostructured materials) to permeate easily and deeper into cellulose fibers than micrometer-sized polymeric PVAMBs. However, this fact could represent a disadvantage in cleaning paper surface from adhesives as it allows spreading of pollutants on the paper sample. Moreover, swelling^45^ and affinity^46^ of cellulose fibers interacting with organic solvents and surfactants have been documented, with the risk of not being able to remove them completely.

### Characterization of the PVAMBs and Paper Interaction Using the Most Efficient Cleaning Protocol

A detailed analysis was performed on the most effective treatment (PVAMB solution directly on paper, US for 2 min, and dabbing with PVA gel) concerning the interaction between PVAMBs and adhesive, as well as the effects of cleaning on paper samples in terms of paper morphology and condition.

### PVAMBs and Paper Interaction: Adhesive Removal Efficacy

**Figure 3:**
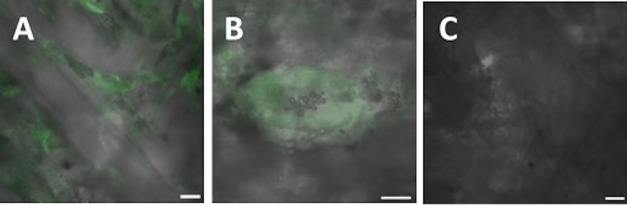
Epifluorescence micrographs of the adhesive adhered on the paper sample (A) before treatment; (B) after PVAMB addition and before using US; and (C) the same (B) spot after using US and cleaning process. Scale bar: 20 μm.

**Figure 4:**
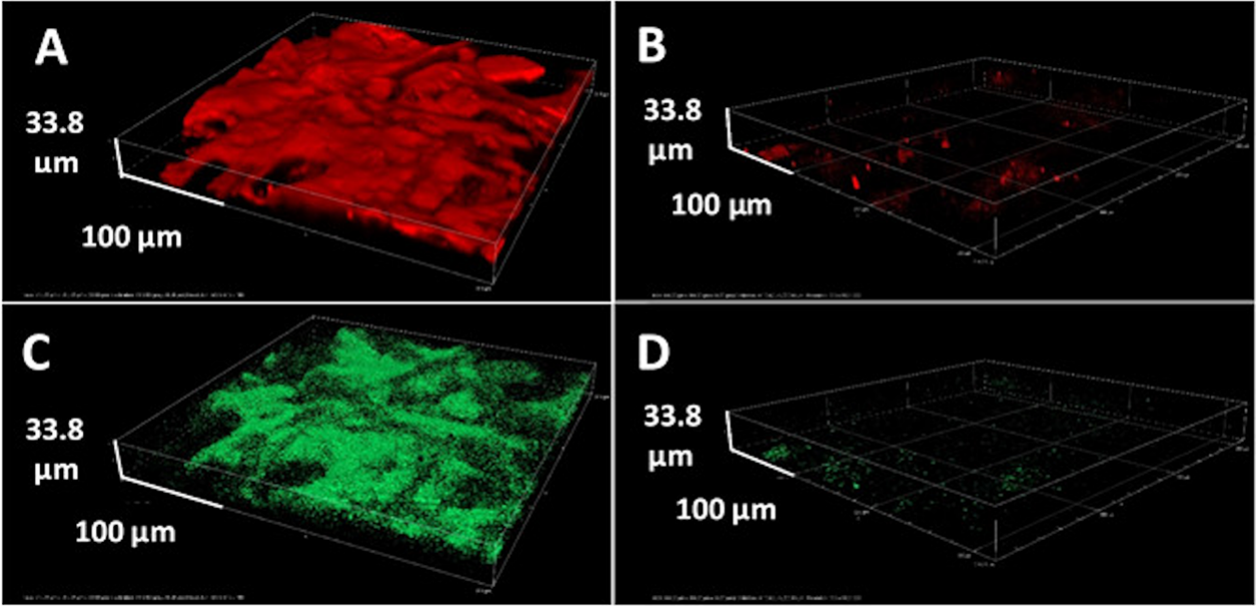
3D confocal laser scanning microscopy reconstruction of the PSAT residuals on an uncleaned paper sample before treatment (A, C) and after treatment using US and cleaning processes (B, D). The treatment was performed from the top, and as can be noticed from the images, the only adhesive residues are left on the bottom of the paper sample. The top images are recorded in the red channel, while the bottom images are recorded in the green channel.

Additional information on the interaction of PVAMBs and paper has been obtained by confocal laser scanning fluorescence microscopy, using fluorescent PVAMBs labeled with an FITC dye. As shown in Figures 3 and 4, aged adhesive is intrinsically fluorescent, and the fluorescence intensity in the red and green spectral regions of the oxidized polymer is not negligible with respect to the fluorescence intensity of polystyrene.^41^

After their addition, PVAMBs tend to locate preferentially on the adhesive-coated part of the samples (Figure 3B). After US treatment and dabbing with PVA gel, the fluorescence intensity of the adhesive was much lower (confirming its removal). For clarity, images related to FITC-labeled PVAMBS on paper samples without adhesive are reported in Figure S6. As shown, the eventual presence of PVAMB residues could be easily visualized. We noted at the end of the treatment, in the water solution removed by the PVA gel, the presence of an amount of PVAMBs (∼35%) transformed into water capsule by the continued solicitation of US^6^ (Figure S7).

Moreover, based on the 3D confocal reconstruction shown in Figure 4, the thickness of adhesive over the paper sheet is about 30 μm and the PVAMBs and US combined treatment was able to remove the traces of adhesive along this depth almost completely.

Summarizing, we assume that the cleaning of the adhesive entrusted to the cavitating microbubbles is effective and progressive due to an effective interaction of the PVA shell of the microbubbles on the rough profile of the area to be treated. This hypothesis is in line with the new cleaning strategies aimed at using elastomeric microparticles to maximize contact at the interface with rough surfaces subjected to cleaning.^15^

In this frame, we also provided contact mode AFM topography images and roughness analysis of the adhesive-coated paper (before and after the treatment) and the one without adhesive coating (Figure S5). Profilometry analysis indicates that micrometer-deep surface depressions, several microns wide, are present in the paper coated by adhesive, which, as expected, are compatible with or larger than the size of our microbubbles. The morphologies highlight a remarkable change after treatment, which is compatible with the removal of surface adhesive layer. Root-mean-square roughness analysis reveals in fact values of ∼460 and ∼340 nm for paper samples with adhesive before and after cleaning, respectively, indicating that the treatment produces a reduction in the surface roughness of the paper coated by the adhesive.

It is interesting to note that, as shown in Figure 3B, fluorescent PVAMBs in water solution are localized only on the adhesive, recognizable by its intrinsic fluorescence. After cleaning, fluorescence due to both PVAMBs and adhesive disappears (Figures 3C and 4B).

These results indicate that PVAMBs allow a localized cleaning action on the adhesive only and that no detectable PVAMB residues remain on paper (as shown also by experiments reported in Figure S6).

The absence of detectable residues of PVAMBs has also been confirmed by HPLC experiments in methanol. The chromatogram relative of the methanol extract of PVAMB sample shows two sharp and two large peaks at 9.5–10 min and at 4–8 and 14.5–18 min, respectively, indicating the dissolution of the PVAMBs in methanol. This dissolution does not occur in water, as the corresponding chromatogram is flat everywhere in the run (data not shown). The chromatographic profiles of the methanolic extracts of modern adhesive alone or on modern paper showed two pronounced peaks, at 3.8 and 5.2 min, and a hump around 8 min. The intensity of these peaks decreased by about 67% after cleaning with PVAMBs, confirming the efficacy of the proposed treatment.

### Evaluation of the Overall Cleaning Efficacy

**Figure 5:**
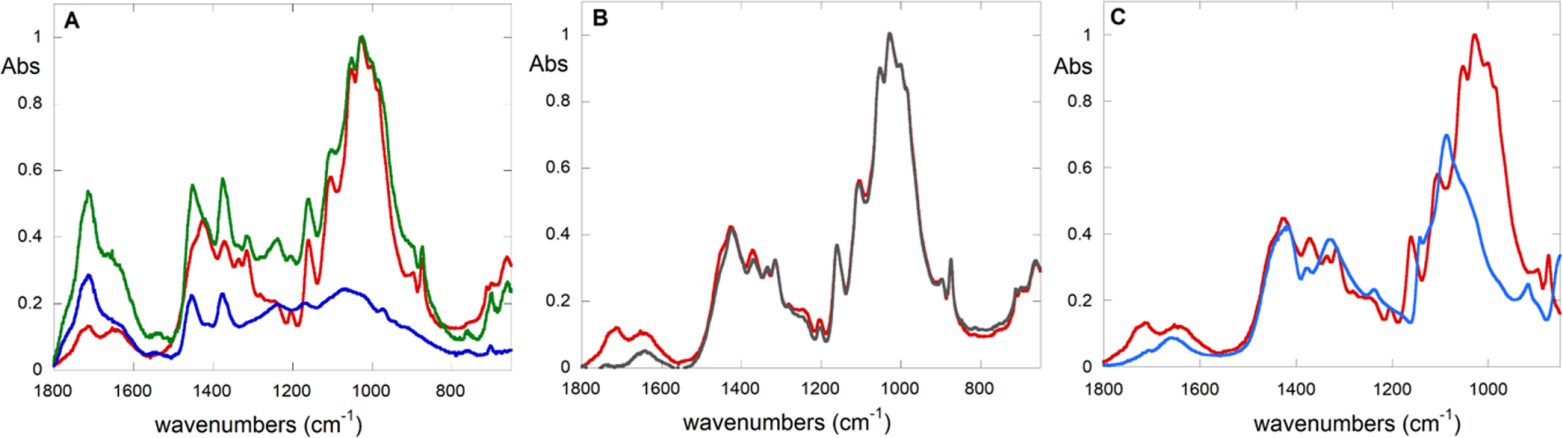
FTIR spectra of (A) uncleaned (green) and cleaned (red) paper samples and of the adhesive (blue); (B) cleaned paper (red) and a paper sample without any adhesive (gray); and (C) cleaned paper (red) and PVAMBs (light blue).

ATR-FTIR analysis of the paper before and after cleaning (Figure 5) clearly shows the decrease in the bands assigned to the adhesive localized principally at about 1730 cm^–1^ due to C=O groups and around 1450 and 1370 cm^–1^ (due to CH_2_ and CH_3_ bending) as well as bending of aromatic =C–H and C=C groups of polystyrene at about 750 and 699 cm^–1^, respectively (see also Figures S3 and S4). Furthermore, a comparison (Figure 5B) between the ATR-FTIR spectrum of the treated sample (red) and that related to the standard sample of the same paper (gray) shows that the two spectra are practically superimposable, apart from small differences due to adhesive residues or cellulose degradation products (at 1450, 1370, and 1240 cm^–1^).

The absence of intensity changes of the 905 cm^–1^ band, mainly due to deformation modes of CCO, COC, CCH, OCH, and stretching vibrations involving C5 and C6 atoms, indicating a change in the amount of amorphous or crystalline structures of cellulose in the sample, suggests that the morphology of paper samples has been preserved and has not been damaged by the treatment.^21,47,48^ Furthermore, no bands attributable to PVAMBs are present in the spectrum of the cleaned paper (Figure 5C), confirming that, above the FTIR detection limit, residues from PVAMB fragmentation after treatment are not present.

Remarkable differences were observed between cleaned paper and paper without adhesive in the 1800–1600 cm^–1^ region of their FTIR spectra. Such differences could be ascribed to adhesive residues and to the presence of absorption peaks due to cellulose degradation byproducts, containing carboxyl and carbonyl groups with absorption in this spectral region.^49^

Chemical aging of paper, leading macroscopically to a loss in optical quality (yellowing), acidity, and a worsening of the mechanical properties are due to strongly interconnected processes, such as oxidation and hydrolysis. These processes cause the formation of various carbonyl groups (like ketones and conjugated diketones), able to absorb visible light (chromophores) and carboxyl groups, responsible for an increase in acidity.^43,49,50^ In addition, the oxidation of the adhesive on paper leads to reactive groups accelerating the degradation of paper. The chromatic variation between the uncleaned paper and the cleaned one is slightly lower than that between the uncleaned paper and the sample without adhesive. This result, together with FTIR ones, could indicate that the cleaning by PVAMBs is not complete (also images in Figures 2 and 5 show that the browning and fluorescence attributable to the adhesive decrease strongly after cleaning, but it does not disappear). Nonetheless, this result could also be ascribable to cellulose degradation.

The evidence that paper is degraded due to the presence of adhesives is confirmed also by pH measurements: pH values of 6.4 and 6.0 ± 0.2 are measured in uncleaned paper with adhesive and in paper with adhesive after cleaning, respectively (pH of plain adhesive is 6.2 ± 0.2), whereas a pH of 7.2 ± 0.2 is recorded in paper without adhesive. As the pH values of PVA gel and PVAMBs solution are pH = 7.0 ± 0.1 and 6.3 ± 0.1 respectively,^12,37^ the acidity increase after cleaning can be attributed to the exposure of degraded paper regions due to adhesive removal.

Chromatography (Figure S8A,B) allowed us to determine the co-presence of both adhesive and cellulose byproducts in uncleaned samples. HPLC results, performed to determine the salts of the several carboxylic acids arising from cellulose degradation in paper samples, clearly show a very complex pattern (Figure S8A). However, peaks relative to lactic and succinic acids, derived from the degradation of the cellulose, are clearly identifiable at retention times of 5 and 6.5 min. The chromatogram of the treated sample presented a sensible decrease in intensity of these two peaks with respect to the untreated one, proving that the treatment removes acidic paper degradation products. At the same time, chromatograms performed under conditions aimed at highlighting the presence of hydrophobic molecules arising from adhesive before and after treatment with PVAMBs indicates that treatment with PVAMBs determined the removal of adhesive (Figure S8B). Importantly, in agreement with FTIR measurements, HPLC results confirmed that, after the treatment, degradation processes potentially highlighted by the presence of byproducts in the chromatograms were not detected.^12,23^ Also XRD data support this finding as the structure of cellulose remains intact after the cleaning treatment (see below).

### Evaluation of US Effect on Paper

The frequency of the US used (1 MHz) is the best trade-off between high-penetrating low-frequency (tens of kilohertz) US treatments reported in the literature,^51,52^ which warns about the mechanical damage they produced, and higher-frequency (∼tens of megahertz) US characterized by a very low penetration and potentially causing thermal damage onto cellulose fibers due to remarkable US absorption. Finally yet importantly, 1 MHz US is adequate to allow a nonresonant oscillation of the PVAMBs^28^ (i.e., small oscillations, preventing a violent and immediate breaking of the shell of the entire irradiated population),^53^ enabling the exploitation of the MB cavitation along the time of treatment.

**Figure 6:**
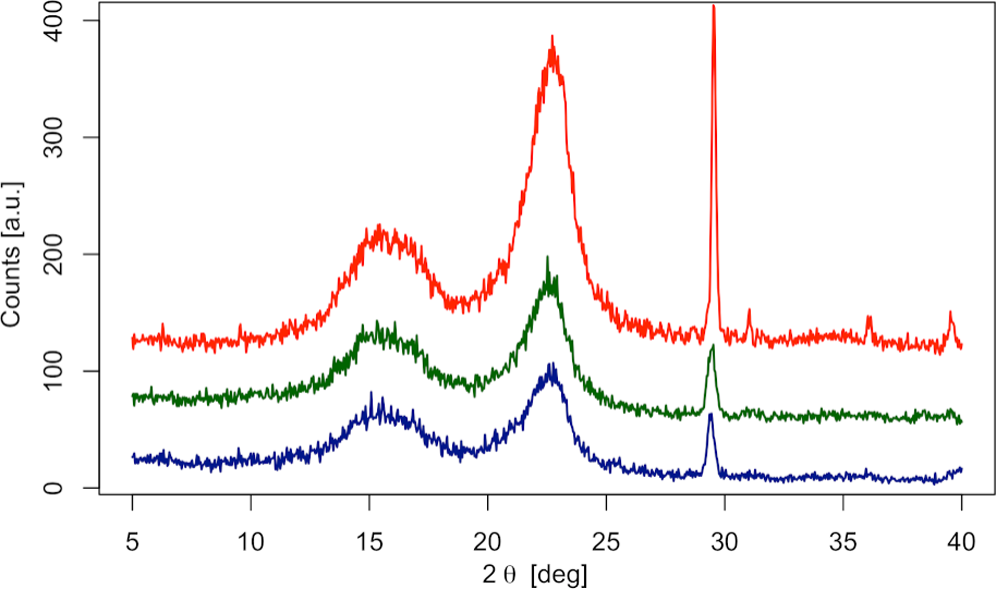
Diffraction pattern of paper without adhesive (red), paper with adhesive before cleaning (green), and paper with adhesive after cleaning (blue).

In this context, the XRD analysis performed under the same experimental conditions for all of the paper samples (paper without adhesive, paper with adhesive, cleaned paper) showed the presence of the same peaks, as highlighted in Figure 6. These common peaks were attributed to the structure of the “native cellulose” (PDF card no. 3-289), that is, cellulose I mainly type β. In particular, looking at the family of diffraction peaks in the range 10–25°, the first broadened peak is normally assigned to the (101̅) plane and the second most intense peak is assigned to the (002̅) plane, the latter representing the crystalline part of the cellulose.^54,55^ Furthermore, the sharp peak before 30° is typically assigned to the presence of calcite (PDF card no. 5-586) and/or gypsum (calcium sulfate dehydrated). The crystallinity index (CI) of all three samples was determined following the methodology of Segal and co-workers,^56^ which is based on the difference of intensity between the two peaks assigned to cellulose as expressed in the following formula:
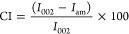
1where *I*_am_ is the intensity of the XRD signal due to the “amorphous” part of cellulose (i.e., (101) and (101̅) reflections), while *I*_002_ is the intensity of the crystalline part.

In particular, XRD analysis (Figure 6) confirms that the most impacting degradation of paper occurs when adhesive is present, as indicated by the comparatively lowest intensity of bands in the diffraction pattern as well as the slightly lower crystallinity index. The CI indeed showed a reduction from 0.8 to 0.7 between paper without adhesive and paper with adhesive before cleaning, while the crystallinity index remained around 0.7 for the cleaned sample, proving that the proposed treatment does not affect the cellulose crystallinity.

This result was corroborated by tensile test analysis performed on paper without adhesive before and after the cleaning treatment. Stress at break values on these samples were strongly comparable (14 ± 2 MPa in both cases); similar results were obtained for strain at break values (in percentage: 2.9 ± 0.4 and 3.0 ± 0.5 for untreated and treated samples, respectively). These data show that the PVAMBs and US treatment do not provide any damage to the paper. Indeed, the use of US on cellulose-based artworks could be discarded considering that a method to depolymerize cellulose is based on US.^57,58^ This apparent weakness in our strategy can be easily disproved by considering the differences in US power, frequency and application time between the protocol proposed in this work and the ones used for cellulose degradation. Moreover, US are applied by a medical device with a working frequency of 1 MHz and low amplitude (the maximum pressure value delivered by the center of the transducer surface is estimated to be about 0.4 MPa, as measured by a hydrophone).^59^ This means that the stable cavitation effects of PVAMBs sufficient to clean and remove the adhesive from the surface fall significantly below the resonant frequency of the PVAMBs. This results in small PVAMBs oscillations, thus avoiding the PVAMBs to be cracked by US^60^ and avoiding excessive microstreaming flows and cavitation hotspots, the latter being the main mechanisms underlying the damages on cellulose.^57,58^ XRD (Figure 6) and tensile tests, as well as ATR-FTIR (Figure 5) results, confirm our thesis since no changes in cellulose crystallinity or in its mechanical properties were observed.

Summarizing, in this article, we propose a novel simple, safe, and “green” methodology for the removal of the aged isoprene-styrene copolymer used as an adhesive on modern paper. It should be noted that the use of PVAMBs has two major advantages: (i) they are biocompatible (already approved and used in medical applications), thus supporting safety for operators and protocols that do not involve the use of solvents. This is an improvement with respect to works reported in the literature,^9,24,27^ in which organic solvents (potentially harmful for operators) are used. (ii) Their diameter is on the micrometric scale (see Figure S1), compatible with paper roughness and porosity (Figure S5).^23^ It is interesting to note that, as shown in Figure 3B, fluorescent PVAMBs in water solution are localized only on the adhesive, recognizable by its intrinsic fluorescence. After cleaning, fluorescence due to both PVAMBs and adhesive disappears (Figures 3C and 4B). These results indicate that PVAMBs allow a localized cleaning action on the adhesive only and that no detectable PVAMB residues remain on paper. In this respect, besides the visual inspection on confocal microscopy, also ATR-FTIR and HPLC results confirm the absence of residual PVAMBs on the treated sample.

Finally, the presence of an amount of PVAMBs (∼35%) transformed into water capsule by US (Figure S7) deserves dedicated attention in a future work, to understand if these capsules can play a role in absorbing and retaining compounds such as cellulose hydrolysis products, similarly to PVA hydrogel.^12,13^

## Conclusions

In this work, we propose the use of PVA-based microbubbles coupled with ultrasounds in an almost unexplored field, that is in the cultural heritage area, i.e., for the removal of adhesive from paper. Based on our previous works assessing the efficacy of PVA-based hydrogels as cleaning tools for modern paper, we have looked for new agents able to remove synthetic polymeric materials such as pressure adhesive without involving toxic solvents. PSAT is a material prone to degradation and very difficult to remove in a safe manner due to their chemical aspecificity. PVA microbubbles have the key features to be suitable cleaning agents: they are suspended in aqueous solution, biocompatible, and already used in medicine. Moreover, thanks to their micrometric dimension, they can penetrate into paper pores, and due to their composition and dynamics under the action of US, they are able to remove adhesive particles in just a few minutes. Fluorescence confocal microscopy and DLS were used to characterize the PVAMBs and their adhesive removal action. Spectroscopic, chromatographic, and pH measurements showed the efficacy of the cleaning procedure, leading to an increase in the optical quality of paper. Finally, XRD experiments and tensile tests assessed the lack of damage on paper due to treatment.

## Supporting Information Available

Confocal microscopy images of FITC-labeled PVAMBs and related Gaussian fit of the diameter size distribution of the PVAMBs; FTIR spectra and HPLC chromatograms of uncleaned and cleaned PVAMB samples (PDF)

## Abbreviations Used

UCAs: ultrasound contrast agents
PSAT: pressure-sensitive adhesive tape
US: ultrasounds
PVA: poly(vinyl alcohol)
tel-PVA: telechelic PVA
PVAMBs: poly(vinyl alcohol) microbubbles
ATR-FTIR: attenuated total reflectance–Fourier transform infrared
XRD: X-ray diffraction
FITC: fluorescein isothiocyanate
DLS: dynamic light scattering
AFM: atomic force microscopy
HPLC: high-performance liquid chromatography
AR: adhesive ratio
C.I.: crystallinity index
