# Supplementary material for: Ultrasound-Stimulated PVA Microbubbles for Adhesive Removal from Cellulose-Based Materials: A Groundbreaking Low-Impact Methodology
Source: ACS Appl Mater Interfaces. 2021 May 14;13(20):24207–17. doi: 10.1021/acsami.1c01892 (PMC8289177; doi:10.1021/acsami.1c01892)
Supplement: Supplementary file 1 — am1c01892_si_001.pdf [file am1c01892_si_001.pdf]

Supporting Information

Ultrasound stimulated PVA-Microbubbles for Adhesive Removal from Cellulose-based Materials: A Groundbreaking Low Impact Methodology

Alessia D'Andrea[†‡], *Leonardo Severini*[†‡], *Fabio Domenici*[†*], *Sultan Dabagov*[§ £ &], *Valeria Guglielmotti* [§ ǂ], *Dariush Hampai*[§], *Laura Micheli*[†], *Ernesto Placidi*[$], *Mattia Titubante*[†], *Claudia Mazzuca*[†*], *Gaio Paradossi*[†], *Antonio Palleschi*[†]

[†] Department of Chemical Science and Technologies, University of Rome "Tor Vergata"
Via della Ricerca Scientifica 1, 00133, Rome, Italy
[§]INFN-LNF, XLab Frascati Via Enrico Fermi 54, 00044 Frascati (RM), Italy
[ǂ] University Guglielmo Marconi, Via Plinio 44, 00193 Rome, Italy
[£] RAS P.N. Lebedev Physical Institute, Leninsky pr 53, 119991 Moscow, Russia
[&] National Research Nuclear University MEPhI, Kashirskoe Sh. 31, 115409 Moscow, Russia
[$] Department of Physics, Sapienza University of Rome, P.le Aldo Moro 2, 00185, Rome, Italy

*Corresponding Authors e-mails: claudia.mazzuca@uniroma2.it; fabio.domenici@uniroma2.it

[‡] These authors contributed equally

Supplementary Figures and Scheme

EXPERIMENTAL METHODS

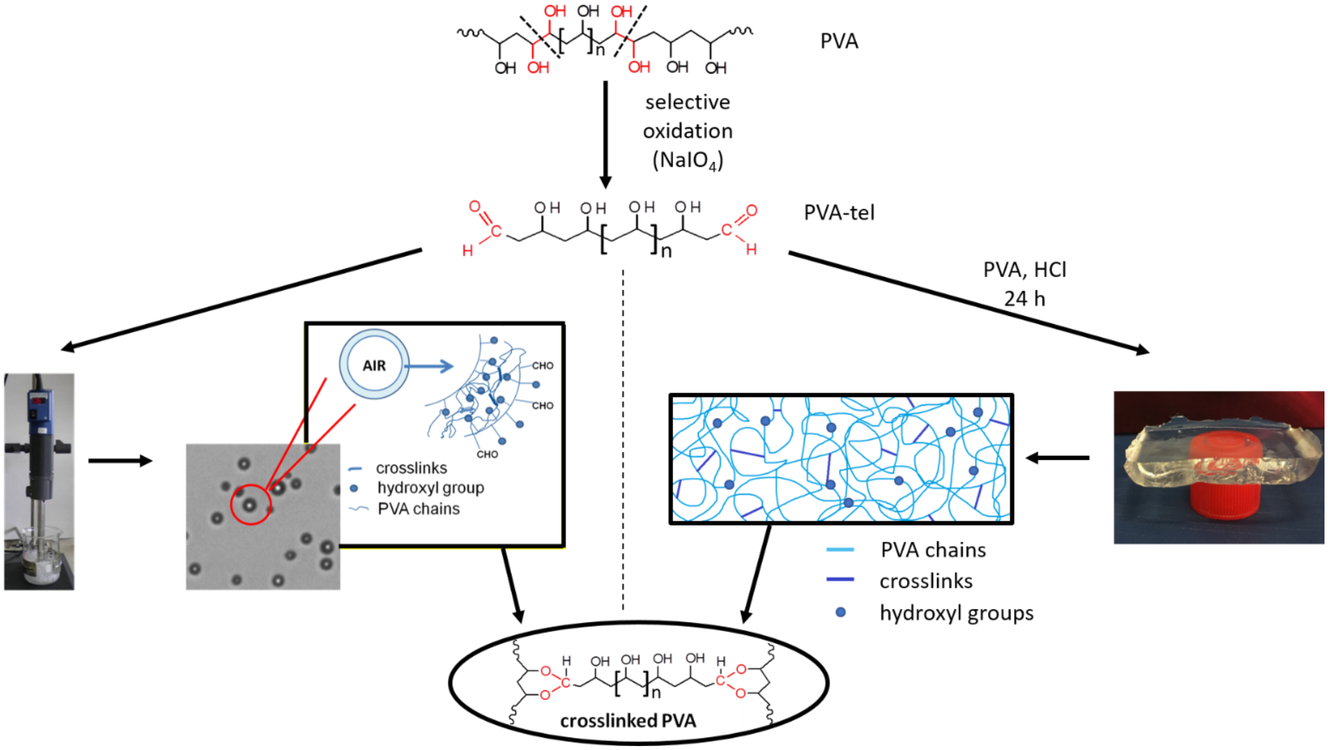

Scheme 1. Synthesis steps of PVAMBs and PVA hydrogel

RESULTS AND DISCUSSION

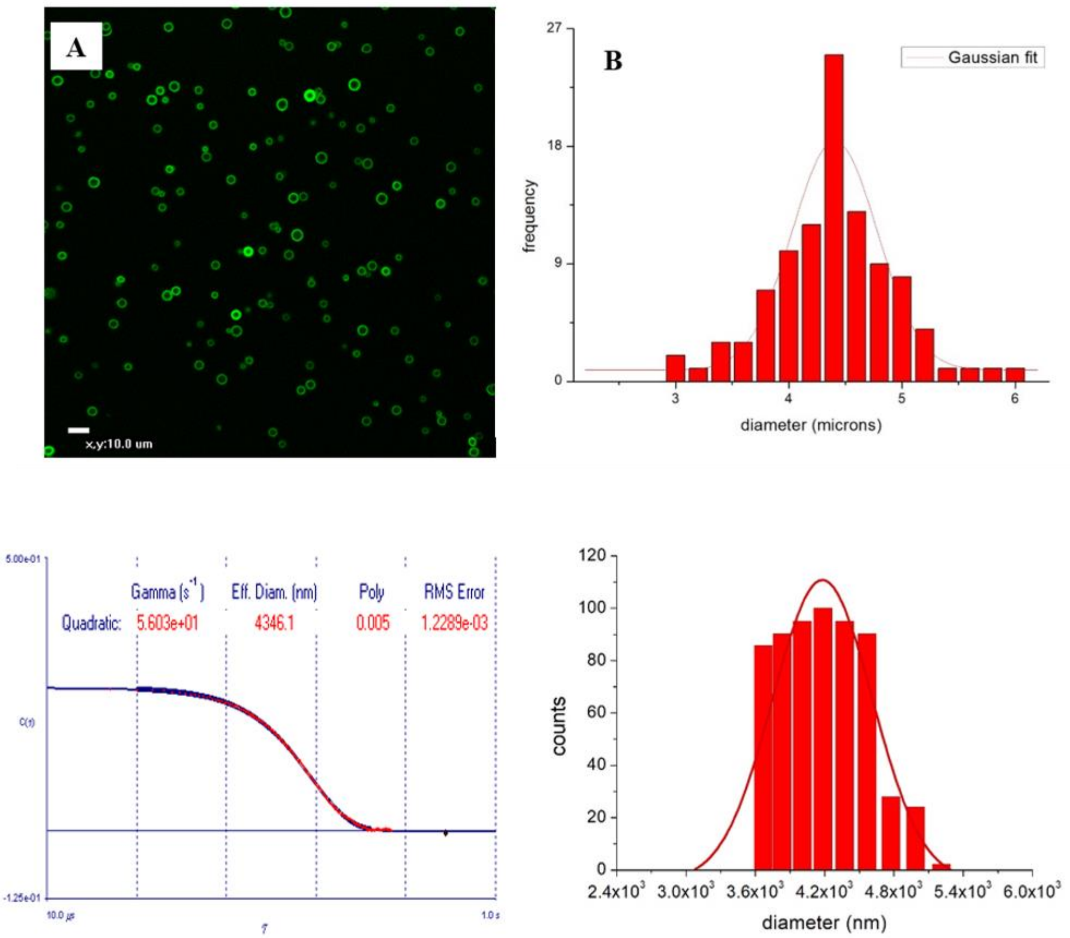

Figure S1. (A): confocal microscope images of FITC-labelled MBs; (B): histograms and related Gaussian fit of the diameter size distribution of the PVAMBs obtained by confocal microscope images. The mean diameter of microbubbles (PVAMBs) assessed through the confocal microscopy pictures is 4.4 ± 0.3 microns. (C): dynamic light scattering measurements; (D): The mean diameter of microbubbles (PVAMBs) assessed through CONTIN algorithm applied to DLS data is 4.2 ± 0.4 microns.

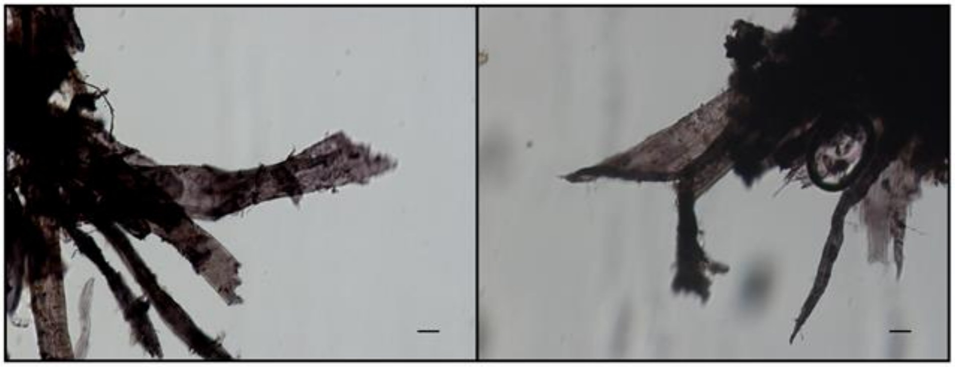

Figure S2. Microscope optical images of fibers of the *notebook* paper, colored with Graff "C" stain. Bar scale: 20 µm; magnification: 20x.

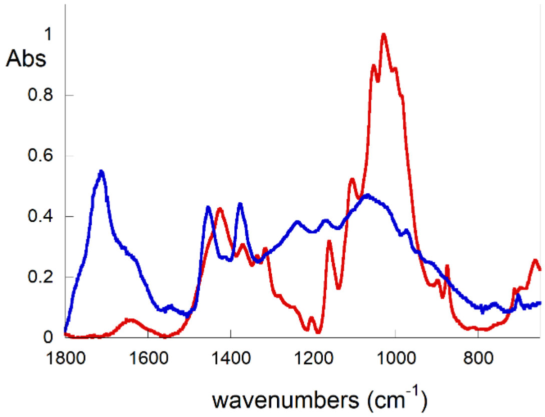

Figure S3. ATR-FTIR spectra of the paper sample (blue) and of the synthetic rubber adhesive (red).

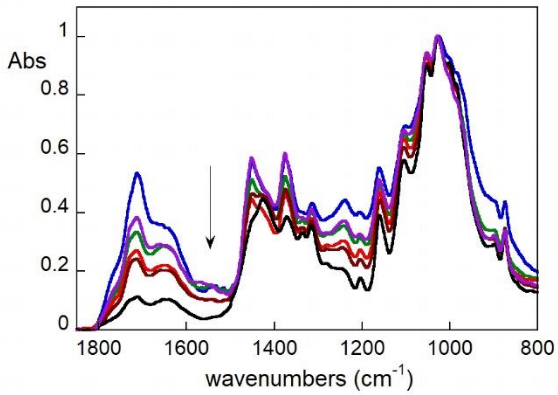

Figure S4. ATR- FTIR spectra of the paper samples containing adhesive: untreated (blue); cleaned with water and ultrasound (US) (2') (violet); cleaned with PVA hydrogel alone (green); cleaned with PVA and then treated with US for 2' (red); cleaned by PVA hydrogels with PVAMBs sputtered on it, then treated with US (2') and finally dabbed with PVA hydrogel (brown); cleaned by PVAMBs spread on paper, then treated with US (2') and finally dabbed with PVA hydrogel (black).

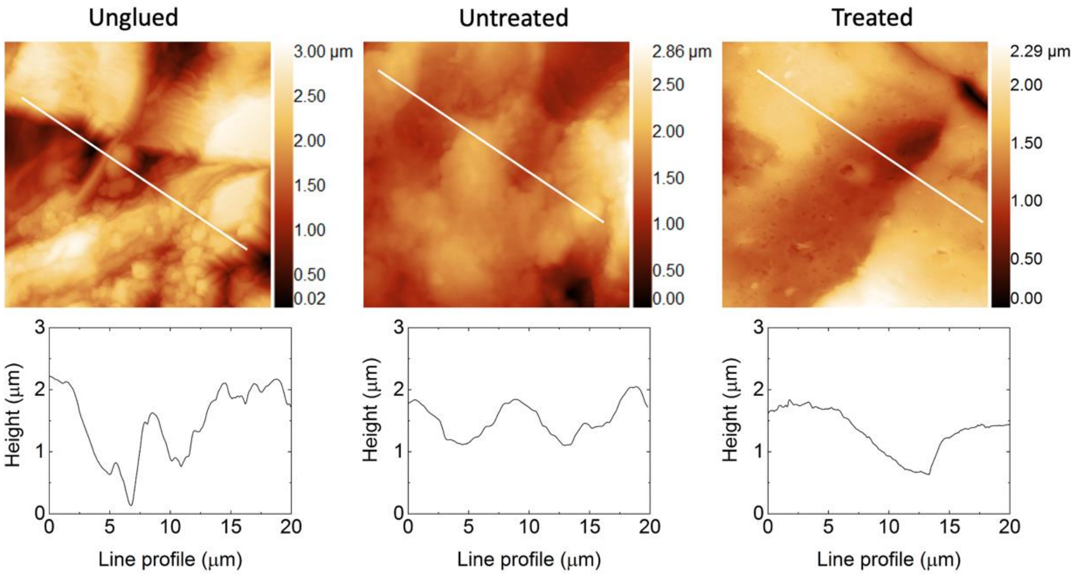

Figure S5. AFM topographies (20x20μm^2) of paper (left): without adhesive; with adhesive before (middle) and after (right) cleaning treatment. Corresponding line profiles are shown below each image.

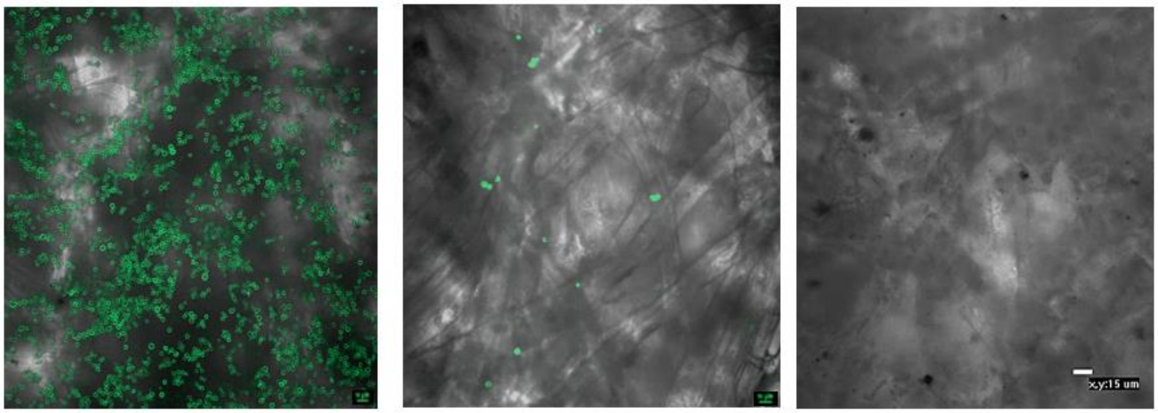

Figure S6. Epifluorescence micrographs of PVAMBs on paper without adhesive before (*left)*; and after treatment in which PVAMBs are (*middle)* almost completely removed (few PVAMBs are left as a reference image) and *(right)* completely removed.

We also provide a picture of what the PVAMBs look like after treatment. A small amount of PVAMBs dispersion has been observed with the microscope after treatment: some of the shells break during the insonation and form capsules, which fill with water and plunge to the bottom of the dispersion. The bottom of said sample is shown in the figure. We estimated that at the end of the treatment about 35% of PVAMBs turned into capsules.

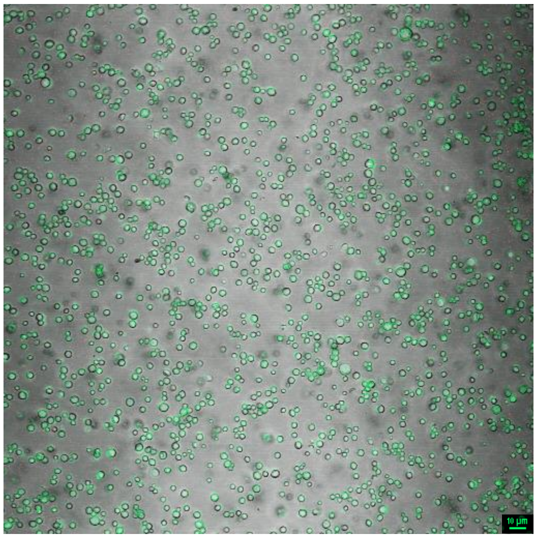

Figure S7. Confocal fluorescence micrograph merged with bright light of a sample of PVAMBs which transformed into water capsules by 2 minutes US irradiation.

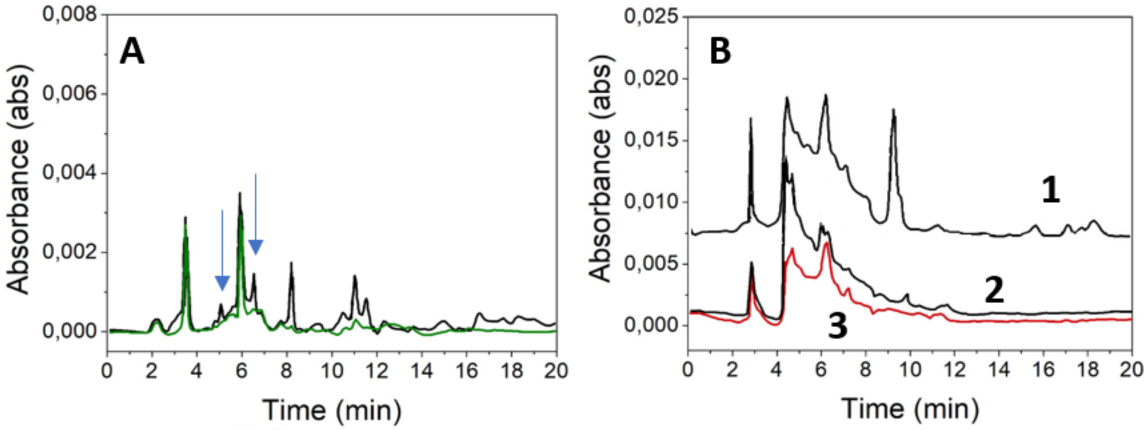

Figure S8. HPLC chromatograms from (A): water extracts of uncleaned *(black)* and cleaned (*green)* paper samples containing adhesive *(peaks at retention times of 5 and 6.5 minutes due to lactic and succinic acids are labeled with blue arrows for clarity);* (B): methanolic extracts of adhesive only *(1),* uncleaned *(2)* and cleaned (*3)* paper samples containing adhesive.
